# Ultrafast laser-induced integrated property–structure modulation of Ge_2_Sb_2_Te_5_ for multifunction and multilevel rewritable optical recording

**DOI:** 10.1515/nanoph-2022-0133

**Published:** 2022-05-17

**Authors:** Kang Zhao, Weina Han, Zihao Han, Xiaobin Zhang, Xingyi Zhang, Xiaofeng Duan, Mengmeng Wang, Yanping Yuan, Pei Zuo

**Affiliations:** Laser Micro/Nano-Fabrication Laboratory, School of Mechanical Engineering, Beijing Institute of Technology, Beijing 100081, China; Beijing Institute of Technology Chongqing Innovation Center, Chongqing 401120, China; Beijing Engineering Research Center of Applied Laser Technology, Beijing University of Technology, Beijing 100124, China; School of Mechanical and Electrical Engineering, Wuhan Institute of Technology, Wuhan 430073, China

**Keywords:** Ge_2_Sb_2_Te_5_, grayscale identification, integrated property–structure modulation, multifunctional and multilevel rewritable storage, ultrafast laser, visual structural color

## Abstract

In this paper, we report an approach for tuning the surface morphology and phase of Ge_2_Sb_2_Te_5_ (GST) by using an ultrafast laser in a one-step process. Four surface micro/nanostructures with specific phase states were sequentially formed by changing the pulse energy: the modified ripple structure, the completely crystallized structure, the ablated nanodots, and the ablated ripple structure. A high correlation existed between the surface micro/nanostructures and their property. Through integrated property–structure modulation, multifunctional optical recording could be achieved by using modified ripples with specific crystallized phase states. The geometric grating morphology caused by the volume shrinkage effect during crystallization enabled modified ripples to exhibit a structural color based on the grating’s diffraction effect. Moreover, the considerable change in the reflectivity of the crystallized area enabled easy grayscale identification. On the basis of the spatially resolved phase-transition threshold effect, the integrated modulation of the geometric nanograting proportion and degree of crystallization was conducted in multilevel states. Notably, different from the fixed ablated surface structures, the printed modified surface structures could be erased and rewritten by controlling its phase state. This paper presents a promising method for producing dynamic tunable metasurfaces, conducting optical anticounterfeiting, and achieving information storage.

## 1 Introduction

Phase-change materials (PCMs) have been developed for nonvolatile and reconfigurable nanophotonic applications. Among PCMs, chalcogenide compounds, particularly Ge_2_Sb_2_Te_5_, have attracted increasing attention for possessing contrasting properties in their various crystallographic phases, high thermal stability, high switching speed, and numerous achievable modulation cycles [1–3]. Regarding the development of micro/nano-fabrication and modulation techniques, many active nanophotonic applications with controllable optical responses by GST, such as color displays, optical memory, and all-optical modulators, have been achieved [4–11].

The precise control of optical responses in functional photonic devices usually depends on the geometric structure and properties of the materials the devices are made of. For example, the original electromagnetic wave steering functionality in so-called metasurfaces is affected by the arrangement of their metallic or dielectric micro/nanostructures (termed as “meta-atoms”); thus, the intrinsic property limits of natural solids can be overcome with considerable freedom. It is highly desirable to dynamically manipulate the optical characteristics of metadevices via external control parameters. Such as, the liquid crystals based reconfigurable based on the reaction of the LC molecules [12]. In particular, a very recent study has demonstrated the dual-function color display using catalytic Mg nanostructures based on the phase transition between Mg and MgH_2_ [13]. Substantial efforts have been focused on developing multifunctional photonic devices by using GST, whose phase states can be selectively controlled through thermal [14], electrical [8], and optical [15] stimuli. Two types of GST structures are used in multifunctional photonic devices. First, GST can be inserted into the plasmonic metasurface as an active medium layer to change the effective refractive index of the dielectric environment, which leads to a considerable shift in the spectral resonance. Under the aforementioned condition, the functional activity of devices is mainly driven by the intrinsic phase properties of GST. Studies have been conducted on optical transmission, absorption and reflection spectra, and spatial light distribution modulation by triggering active GST layers to exhibit multilevel intermediate phase states [16–18]. For example, diverse colors can be modulated on the basis of the interface phase-change mechanism by using an optical coating composed of a GST film with controllable phase states [19, 20]. High-density spatially resolved multilevel grayscale image storage has been realized by stimulating the multilevel crystallization of GST thin films [21]. In addition to material properties, the geometric parameters of constructed micro/nanostructures also affect the functionality of the photonic devices. Thus, GST micro/nanostructures can be constructed as components of optical functional devices, whose structural parameters have a strong influence on their original optical resonance properties. By fabricating GST with designed structures, different functionalities have been achieved. For example, broadband and controllable absorbers or filters that operate in the visible, near-infrared, and mid-infrared ranges have been developed by constructing GST with square or circular nanostructures [22, 23]. Optically switchable reflectivity and transmission resonances by constructing GST with subwavelength nano-gratings [24]; by constructing GST with plasmonic metallic, all-dielectric, or high-refractive-index nanostructure into hybrid nanostructures [25–27].

GST has been successfully used to direct light manipulation. However, they are limited to independent material properties or structural parameter study. Few studies have attempted to achieve the integrated modulation of material and structural properties because of the limitations of processing techniques [8, 14]. The aforementioned modulation may offer a solution for meeting the increasing demand for the manufacturing and tuning of active multifunctional devices. Among the various external stimulation techniques, ultrafast lasers can be used to achieve integrated property–structure modulation for producing user-designed components with multi-material properties and controllable structures. Because of their nonlinear and nonequilibrium effects, ultrafast lasers, which are one of the most advanced manufacturing tools for constructing structures, can be used to fabricate various zero-dimensional, one-dimensional, two-dimensional (2D), and three-dimensional (3D) micro/nanostructures with high quality and flexibility [28]. In particular, ultrafast laser pulses can induce PCMs to enter metastable semi-crystallized states (also termed as intermediated states) with an extremely more sophisticated affected volume [10, 29, 30].

Therefore, in the present research, we experimentally demonstrated that the surface micro/nanostructures, and phase states of GST can be integrated controlled using the ultrafast laser writing technique. Four types of periodic surface structures with different crystallized phase states were produced by deliberately changing the laser parameters. Typically, functionalized colorizing effect in the visible regime was observed for the geographical structural surface based on the diffraction effect of its grating morphology. This effect was accompanied by considerable changes in optical properties with multilevel crystallized phase states. The integrated modulation of the structure and properties of GST enabled multiple functions to be achieved with high flexibility in one step. As a demonstration, multifunctional image storage with multi-brightness structural colors as well as multi-grayscale identification was realized using the modified ripples of GST by carefully controlling its properties and structural geometry. The grating proportion and crystallization degree were controlled on the basis of the laser-induced threshold effect. The properties of the modified ripples were also controlled quantitatively on the basis of the aforementioned effect. This opens up the possibility of new control degrees of freedom and functionaries for the fabrication and modulation of reconfigurable photonic devise in a one-step all-optical process.

## 2 Design and fabrication

**Figure 1:**
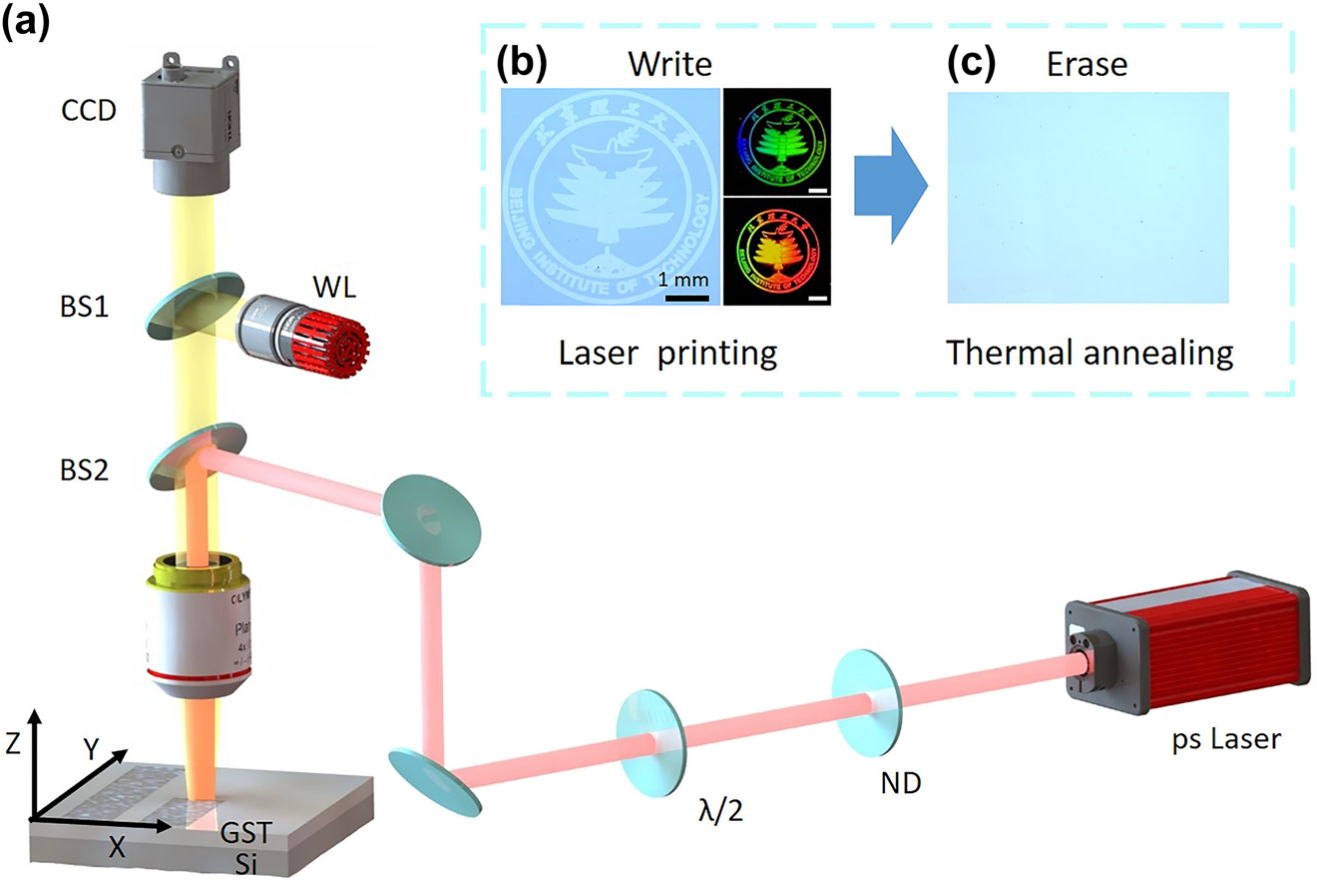
Schematic diagram of the experimental process. (a) Experimental setup for processing with a picosecond laser. The red line represents the laser path, and the yellow path represents the path of imaging light in the coaxial system. (b) Optical micrographs of modified ripple pattern on the GST surface by laser direct writing. (c) Optical micrograph of the patterned sample in (b) after thermal annealing.

The schematic of the experimental arrangement is displayed in Figure 1a. The ultrafast laser source used for processing was a picosecond (ps) laser system (Huaray) that delivers Gaussian-shaped pulses with a pulse duration of 9.3 ps, a central wavelength of 1064 nm, and a repetition rate of 200 kHz. In this research, the laser pulse repetition rate was reduced to 1 kHz by using a pulse picker. Samples were mounted one at a time on a computer-controlled three-axis moving stage (Aerotech A3200). The ps laser pulses were focused on the sample surface through an F-theta lens (*f* = 50 mm). An achromatic half-wave plate and a linear polarizer were used to control the laser pulse energy. Neutral-density filters were employed to enable variable adjustment of the laser pulse energy incident on the sample surface. Another half-wave plate was used to change the polarization direction of the incident laser pulse. A coaxial imaging system consisting of a charge coupled device camera with a white-light source was used for the real-time observation of the fabrication process. Predesigned images were coded using MATLAB and divided into several processing regions according to their gray values. The laser writing routes of specific regions were generated, including the filled density of the line pixels and the power of the laser pulses. Figure 1b depicts the optical micrographs of the ps laser-induced modified ripple pattern on the GST surface, with a grayscale micrograph displayed and colored micrographs displayed. Figure 1c shows that the printed image was erased by thermal annealing.

## 3 Results and discussion

### 3.1 Modulation of the surface micro/nanostructures based on laser-surface plasmon polaritons interference

**Figure 2:**
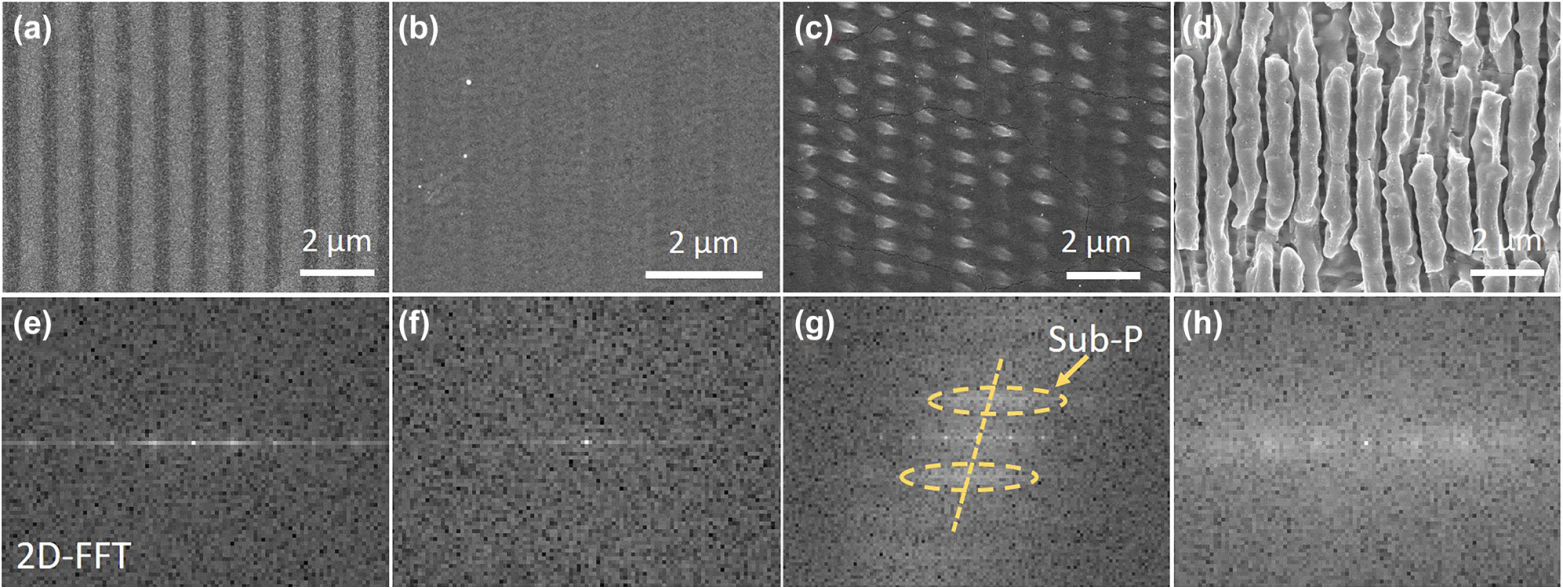
SEM images of the characteristic structures formed during the morphological evolution of GST under irradiation by increasing laser energy. (a) 62.4, (b) 91.2, (c) 155.6, and (d) 173.5 nJ, the scanning speed was fixed at 500 μm/s. Figures (e)–(h) depict the 2D-FFT of the SEM images shown in (a)–(d), respectively.

In the course of laser processing, the interference of laser and surface plasmon polaritons (SPPs) can manipulate the distribution of the electromagnetic field [21, 31], and further realize the manufacturing of the surface micro/nano-structures. Laser induced periodic surface structure (LIPSS) is considered as the most typical self-organized surface structure, generated by the laser–SPPs interference [32–35]. In general, the morphology of LIPPS is determined by the laser–SPPs interference field distribution. It is vital to modulate the field distribution of laser–SPPs interference, thus to modulate the resulted LIPSS morphology. Normally, the direct laser–SPPs interference and structure-assisted laser–SPPs interference are considered as the main two factors affecting the LIPSS fabrication [36]. Thus, the distribution of interference field is mainly affected by the following two factors: (1) laser processing parameters [37]; (2) the feedback effect of pre-existing surface structures [36], and both of them are correlative and interactional. In this study, we prepare periodic surface micro/nano-structures on GST film with controllable morphologies by controlling the pulse energy to modulate the laser–SPPs interference. Figure 2a–d shows the four typical surface structures fabricated on GST between ablation and crystallization: the modified ripple structure, the completely crystallized structure, the ablated nanodots, and the ablated ripple structure. Figure 2e–h shows the 2D fast Fourier transform (2D-FFT) analysis, which confirmed the presence of unique spatial periodicity in these four types of surface structures in Figure 2a and b, respectively.

**Figure 3:**
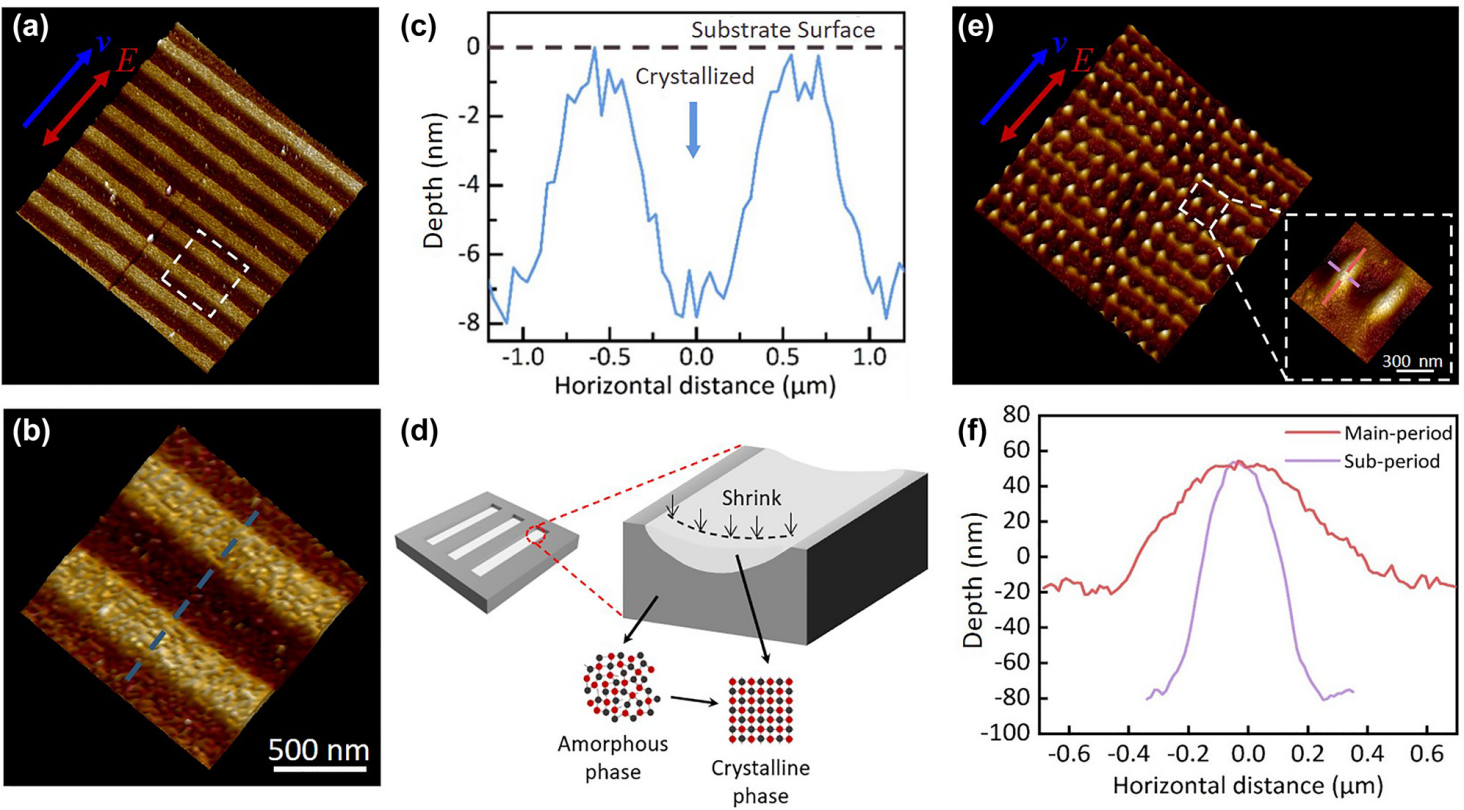
AFM images of the modified ripple structure and ablated nanodots. Figure (a)–(c), and (e)–(f) present the depth and period of the modified ripple structure and ablated nanodots structure, respectively. (d) presents a schematic of the formation of a modified ripple structure with the features of periodic diffractive gratings. The magnified view of the white frame marked in (e) is shown in the inset of (e). (b) is a magnified view of the white frame marked in (a).

Figure 2a shows that the modified ripple structure, composed of amorphous stripe (a-stripe) and crystalline stripe (c-stripe), was generated at *E* = 62.4 nJ. Compared to conventional ablated structures, the formation of modified ripple structure is mainly accompanied by phase-change of amorphous GST without the removal of material. Because of the low pulse energy and the relatively smooth material surface, the interference field was not redistributed, which enabled that modified ripple structure can be directly printed by the direct laser–SPPs interference sub-wavelength energy deposition, and can even be erased (as demonstrated in the following section). This avoids the feedback of structure-assisted laser–SPPs, which is the main reason caused the nonuniform and inconsistence of the structures, as demonstrated in the ablated ripple structures shown in Figure 2d. Thus, the modified ripple structure is highly homogeneous, and the regular distribution of the spatial frequency in 2D-FFT plot (Figure 2e) can also prove it [38]. The profile of the modified ripple structure is shown in Figure 2a and b. A volume shrinkage can be observed during the crystallization, enabled the modified ripple structure a geometrical grooved morphology. As shown in Figure 3c, a shrinkage occurred in the crystallized ripple area, resulting in a maximum height of 8 nm between the initial amorphous GST sample surface and the crystalline surface. We contribute this shrinkage effect to the properties’ changes induced by the laser irradiation, as the schematic shown in Figure 3d. The phase change induces a material density difference between amorphous and crystalline state [39, 40]. Thus, the crystallized regions contained surface depressions, and the modified ripple structure exhibited a spatial fluctuation characteristic of periodic diffractive gratings feature.

When the pulse energy was 91.2 nJ, a-stripes disappeared, and the surface of GST was completely crystallized, as shown in Figure 2b. Figure S1 displays the schematic of the evolution from a modified ripple structure to a completely crystallized structure. With an increase in pulse energy, the width of the c-stripes increased, whereas that of the a-stripes reduced. When the width of the crystalline stripes increased to be close to the periodic size, the adjacent crystalline stripes connected to form a completely crystallized structure on the surface.

Further increasing the pulse energy to ablation threshold, *E* = 155.6 nJ, the ablated nanodots can be observed, as shown in Figure 2c. For the ablated nanodots, in addition to stripes perpendicular to the laser polarization direction, stripes parallel to the laser polarization direction appeared on the surface. Figure 2g shows a crossing distribution of spatial periods, which indicate that there exists a sub-period perpendicular to the main period in ablated nanodots. Thus, it can be seen that the ablated nanodots is a kind of laser-induced orthogonal periodic structures. We defined the fringe structure in the direction of vertical laser polarization as the main-period, and the fringe structure in the direction of parallel laser polarization as the sub-period. Figure 2e and f shows the AFM images of the ablated nanodots. We can observe that the main-period was greater than the sub-period. Moreover, the morphology of each ablated nanodot is different in the two directions. Figure 3f shows that the height and width of the ablated nanodots in the direction of main-period are 130 and 550 nm, respectively, while the height and width in the direction of sub-period are 70 and 900 nm, respectively. To gain deeper insight into the mechanism of the formation of ablated nanodots, we calculated electric field distributions affected by the initially formed gratings (pre-existing surface structure) by using a commercial software (Lumerical FDTD Solutions), as shown in Figure S2. As demonstrated in the simulation, the initial formed surface structure leads to a redistribution of the electric field, resulting the periodic nanodots structures formation. The subsequent ablation occurred primarily at the field enhancement area, which resulted in the formation of the nanodots structure. The formation of the ablated nanodots can be considered as a transition state between the formation of completely crystallized structure and ablated structure. After irradiation with an energy of 173.5 nJ, the surface was damaged, forming the conventional ablated ripple structures, as shown in Figure 2e. The debris, defects, initially formed structures during the ablation produce feedback for the redistribution of interference field [36], leading to a significant nonhomogeneity of the consequent structures. To sum up, the formation of the ablated nanodots and ablated ripple structures conformed to a negative feedback mechanism induced by the interference of structures-assisted laser–SPPs coupling.

**Figure 4:**
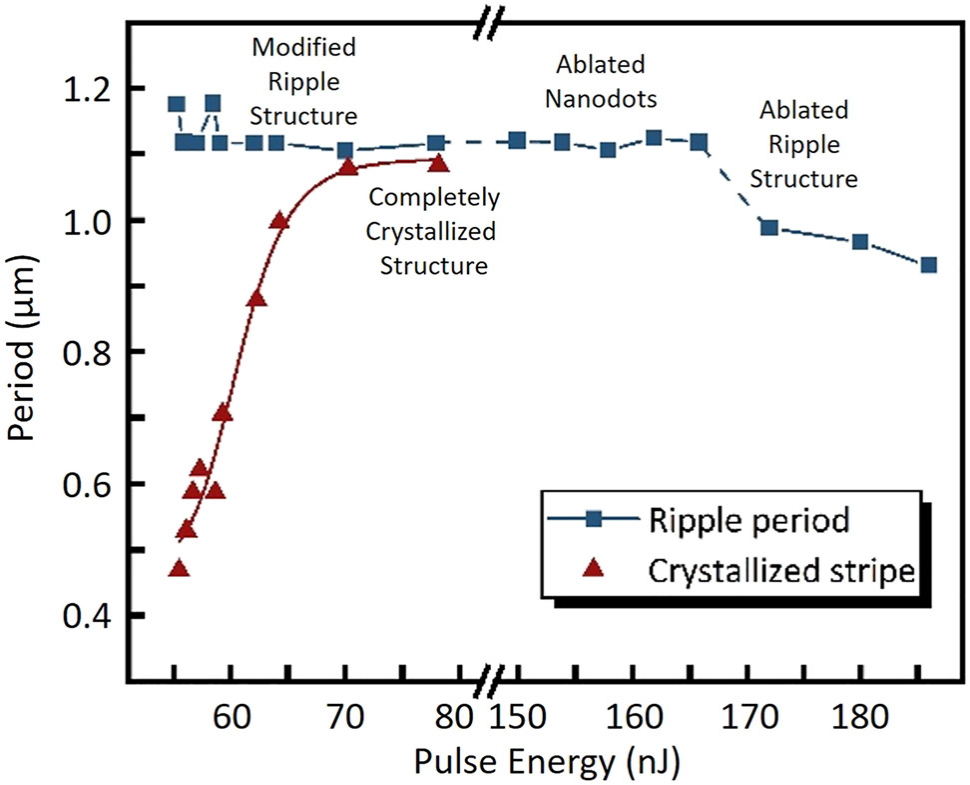
Parameters of the surface structures for different pulse energies.

Figure 4 illustrates the evolution of the period of the four structures formed under different pulse energy values. The periods of the modified ripple structure, completely crystalized structure, and the main period of the ablated nanodots remain nearly unchanged around 1100 nm with different pulse energies. Although ablated nanodots existed a sub-period parallel to the laser polarization direction under the feedback of surface microscopic structures, it did not exert an obvious effect on the main period perpendicular to the laser polarization direction. Thus, the pure laser–SPPs interference mechanism dominates plays a dominant role in the periodicity of modified and weak ablation structures. Therefore, the period remains consistent to the periodic energy distribution caused by the laser–SPPs interference, which is near the incident laser wavelength. Different from the other three structures, the period-decreasing phenomenon originates in the ablated ripple structures and the period decreased with the increasing pulse energy. With the increasing pulse energy, the deepening of the grooves in ablated ripple structures makes its period decrease [36, 41, 42]. Moreover, because of the threshold effect, the width of the c-stripes gradually increased with an increase in the laser energy. When the width of the crystalline stripes increased to be close to the length of the period, the adjacent c-stripes connected to form a completely crystallized structure on the surface. When the pulse energy was marginally higher than the GST ablation threshold, the completely crystallized structure was replaced by an ablated nanodot structure, and the crystalline stripes disappeared.

### 3.2 Analysis of the properties of GST

**Figure 5:**
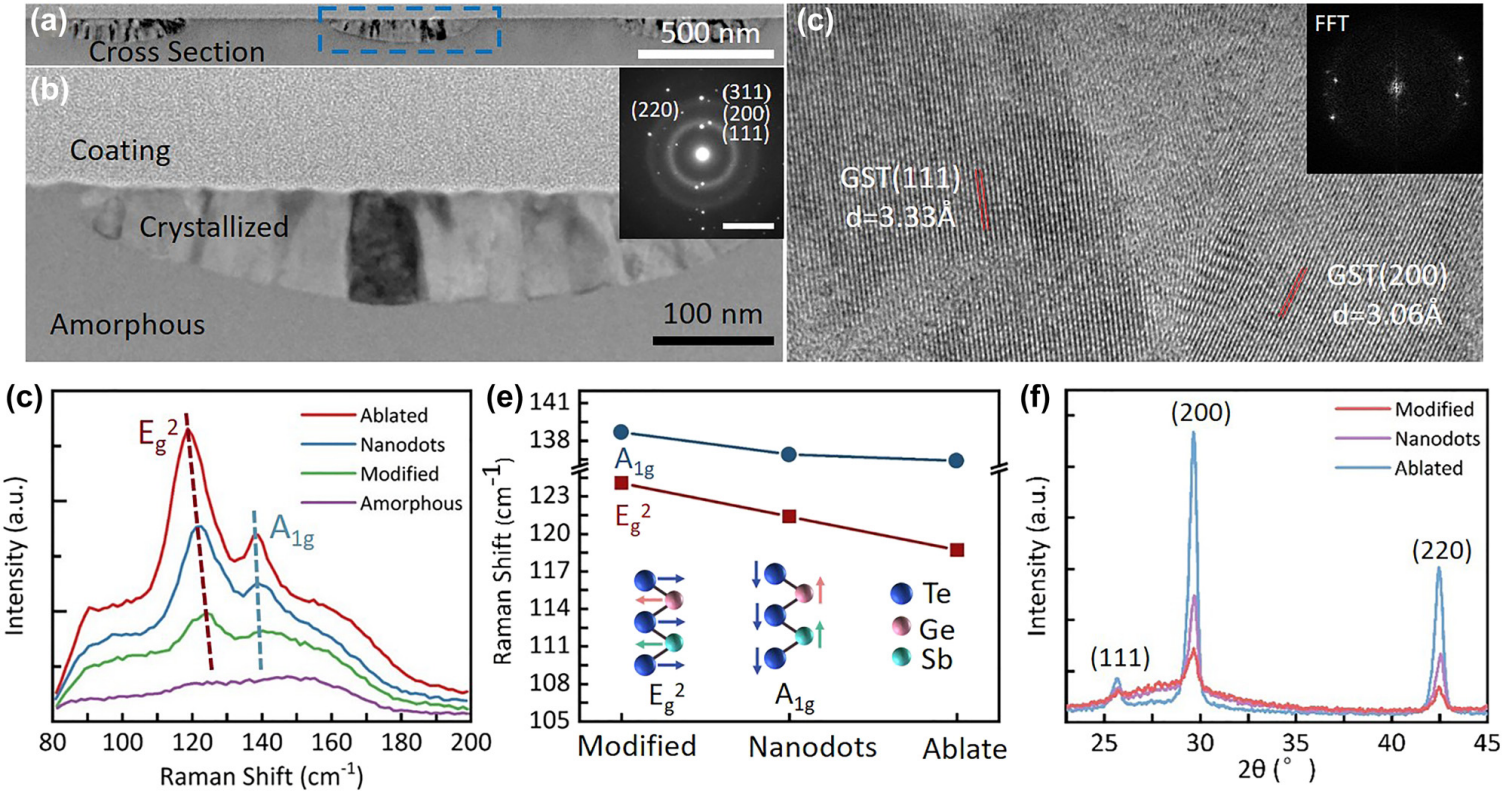
Analysis of the properties of the structured GST. (a) Cross-sectional TEM image of the modified ripple structure. (b) Magnified view of the blue frame marked in (a). The inset in (b) displays the selected area electron diffraction pattern of crystallized regions. (c) High-resolution TEM image of a crystalline–amorphous GST interface. The inset in (c) depicts the fast Fourier transform of the crystallized region. Raman spectra (d) of the initially amorphous GST film, modified ripple structure, ablated nanodots, and ablated ripple structure. (e) Plot of the Raman peak shift versus energy for different surface structures. (f) XRD spectra of the three distinct ps-laser-induced surface structures, which is correspond to Figure 5d.

Material properties changes were accompanied with the structural modulation by the irradiation of ps laser pulses. For GST thin film, its phase changes under laser irradiation determine their macroscopic physical properties. To determine the crystal characteristics of the modified ripple structure, the modified ripple structure was sliced along its periodic direction by using a focused ion beam system to perform TEM measurement. The cross-sectional TEM image was displayed in Figure 5a. It can be observed that the crystallized regions embedded in the amorphous GST were also distributed periodically, and corresponded to the c-stripes of modified ripple structure. Therefore, the modified ripple structure not only is a periodic surface structure but also exhibited the periodic characteristics of a volume-like grating consisting of alternate amorphous regions and crystallized stripes inside the material. Figure 5b depicts the magnified view of c-stripes marked by the blue frame in Figure 5a. The volume-like gratings structures inside the GST film exhibit an inverted parabola morphology, which is similar to that geometrical shrink grating structures shown in Figure 3c due to the crystallization threshold effect. The depth of the central crystallized region was approximately 100 nm, and it decreased gradually from the center to the sides. The inset in Figure 5b displays the selected area electron diffraction pattern of the crystallized regions. The presence of the (111), (200), (220), and (311) peaks in this inset for crystallized GST indicates that face-centered cubic (FCC) nanoparticles of GST were formed [43]. To determine the lattice characteristics in grain growth, a high-resolution TEM image was obtained of a crystalline–amorphous GST interface (Figure 5c). The fast Fourier transform (FFT) of the crystallized regions is depicted in the inset of Figure 5c. This FFT indicates that two types of crystal plane structures exist in the crystallized regions, and these planes are distributed at acute angles to each other. As presented in Figure 5c, the spacing of lattice stripes was calculated to be 3.33 Å and 3.06 Å, which correspond to the (111) and (200) planes of FCC GST, respectively. The aforementioned result indicates that the GST film was crystallized with homogeneous polygonal grains.

Figure 5d depicts the Raman spectra of the initially amorphous GST film, modified ripple structure, ablated nanodots, and ablated ripple structure. The as-deposited amorphous state exhibits a broad peak at approximately 150 cm^−1^, which is considered as the characteristic peak for amorphous GST, and is generally ascribed to the stretching vibrations of amorphous Te–Te bonds [44, 45]. After the laser irradiation, peaks of modified ripple structure, ablated nanodot structure, and ablated ripple structure at 120 cm^−1^ and 140 cm^−1^ can be clearly observed, which are assigned to the E_g_^2^ and A_1g_ modes, respectively [46]. The E_g_^2^ and A_1g_ are mainly caused by the vibration of Ge (Sb) and Te atoms in opposite directions, which mainly affected by both interatomic forces between the Sb and Te atoms and between Ge and Te atoms, respectively (insert in Figure 5e). Usually, the change in Raman peak position can provide more reliable information than the peak intensity. Figure 5e shows a plot of the Raman peak shift of A_1g_ and E_g_^2^ modes versus three surface structures. It can be clearly seen that the frequencies of the A_1g_ and E_g_^2^ modes exhibit a redshift from modified ripple structure to ablated ripple structure. The bond length increment, grain refinement, structure defects increase with increasing pulse energy is considered as the main factors for the Raman peak redshift [46]. Additionally, the thermal stress and phase transformation stress during the crystallization may also play an important role for the redshift. Figure 5f shows the XRD spectra of the three distinct ps laser-induced surface structures. These patterns indicate that crystallinity increases gradually with an increase in pulse energy, as evidenced by the increase in the diffraction intensities of the (200) and (220) reflections, which are the two most intense characteristic XRD peaks in the FCC phase [43]. The aforementioned trend was also observed for the (111) peak. The aforementioned results indicate that the extent of crystallization in the films continually increased as the energy of applied laser pulses increased.

### 3.3 Multifunctional and multilevel rewritable image storage based on integrated property–structure modulation of modified surface structures of GST

The intrinsic differences in the physical properties of the amorphous, intermediate, and crystalline phases of modified surface structures of GST allow the formation of GST films with a 2D grating morphology. Thus, we can realize direct written, property modification, even erase of the GST modified surface structures by using the ultrafast laser pulses in one step, facilitating direct laser writing lithography without need of multi-step nanofabrication technique.

**Figure 6:**
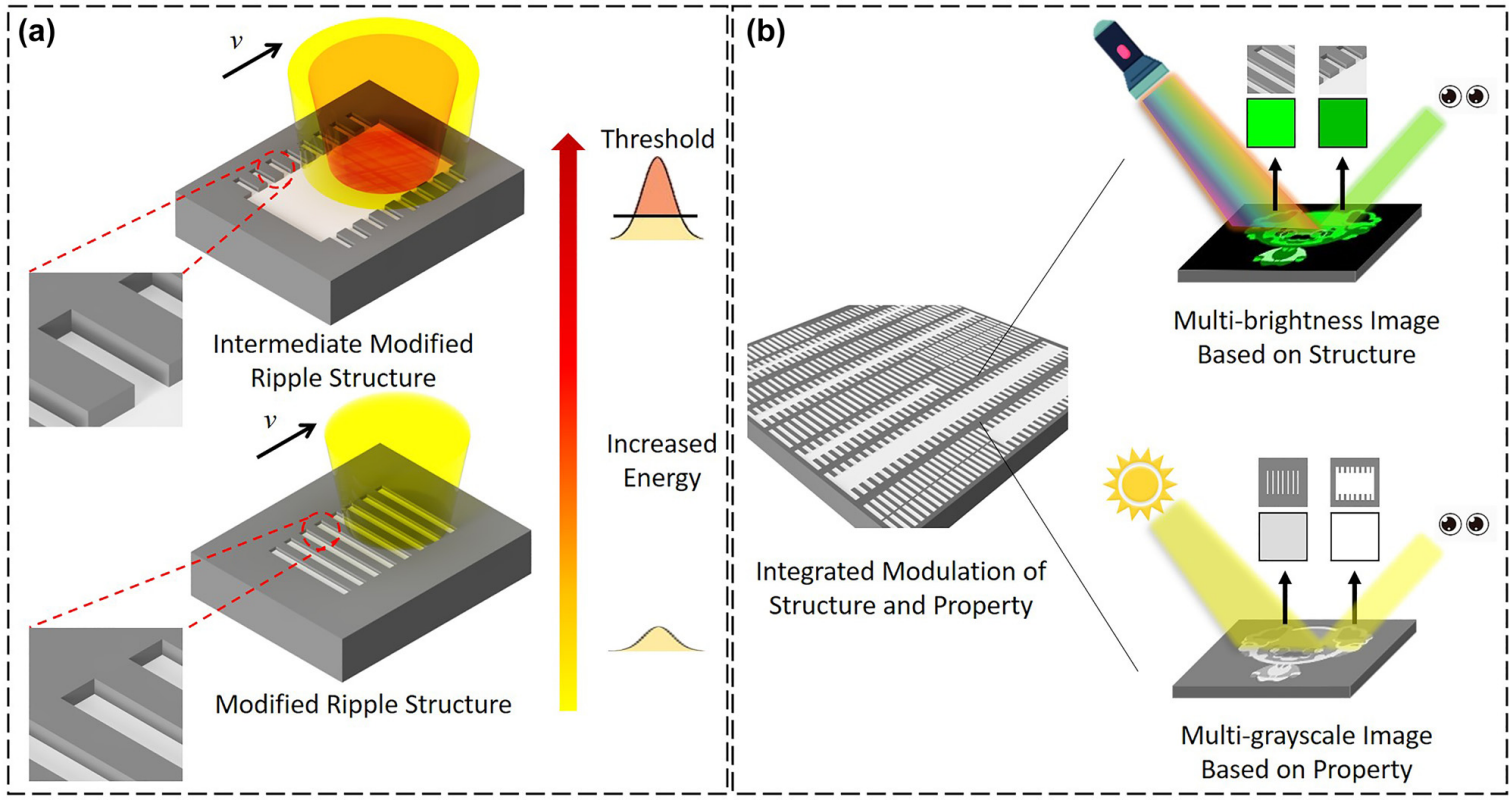
Schematic diagram for multifunction and multilevel rewritable optical recording. (a) Schematic of the integrated modulation of the surface structure and material properties of GST. (b) Sketch of the lighting source-dependent multifunctional and multilevel image storage based on the modified ripple structures, which is observed under illumination of strong light and natural light.

Studies have widely adopted GST in information storage devices [10, 21, 45]. Most of these studies focused on the storage on grayscale optical data, with each grayscale level being dependent on the optical property contrast between different phases. Conventionally, optical properties can be tuned by structural geometries as well as the materials’ property. For example, geometrical 2D grating exhibits iridescent structural colors according to their diffraction effect. However, structure-based optical functionalities depend on the fabrication techniques used and cannot be modulated after structure formation is completed. The GST modified surface structures with controllable property-structure characteristic gives the possibility to written, manipulate, and even erase it with high flexibility. As an application example, we showed a multifunctional (color and grayscale identification) and multilevel (multi-brightness and multi-grayscale) image storage by exploiting the modified surface structures with specific crystallized phase states. The integrated modulation of the geometric parameters of the grating (structural proportion) and the crystallization degree could be controlled by varying the irradiated laser pulse energy according to the threshold effect (Figure 6a). The colorizing phenomenon, whose brightness varied with the proportion of the modified ripple structure, occurred because of its grating-like morphology; moreover, the concomitant completely crystallized area ratio and crystallized degree integrated modulation makes the irradiated area with multilevel grayscale identification (Figure 6b). Notably, the grating structure exhibits iridescence due to the angle-dependent diffraction when the lighting source is strong. This is because the height of the 2D grating is relatively small, which dramatically lessens its diffraction intensity. Thus, upon the illumination of natural light, for example the sunlight, the structural color cannot be recognized and renders the surface with grayscale identification. The detailed demonstration is performed in the following section.

**Figure 7:**
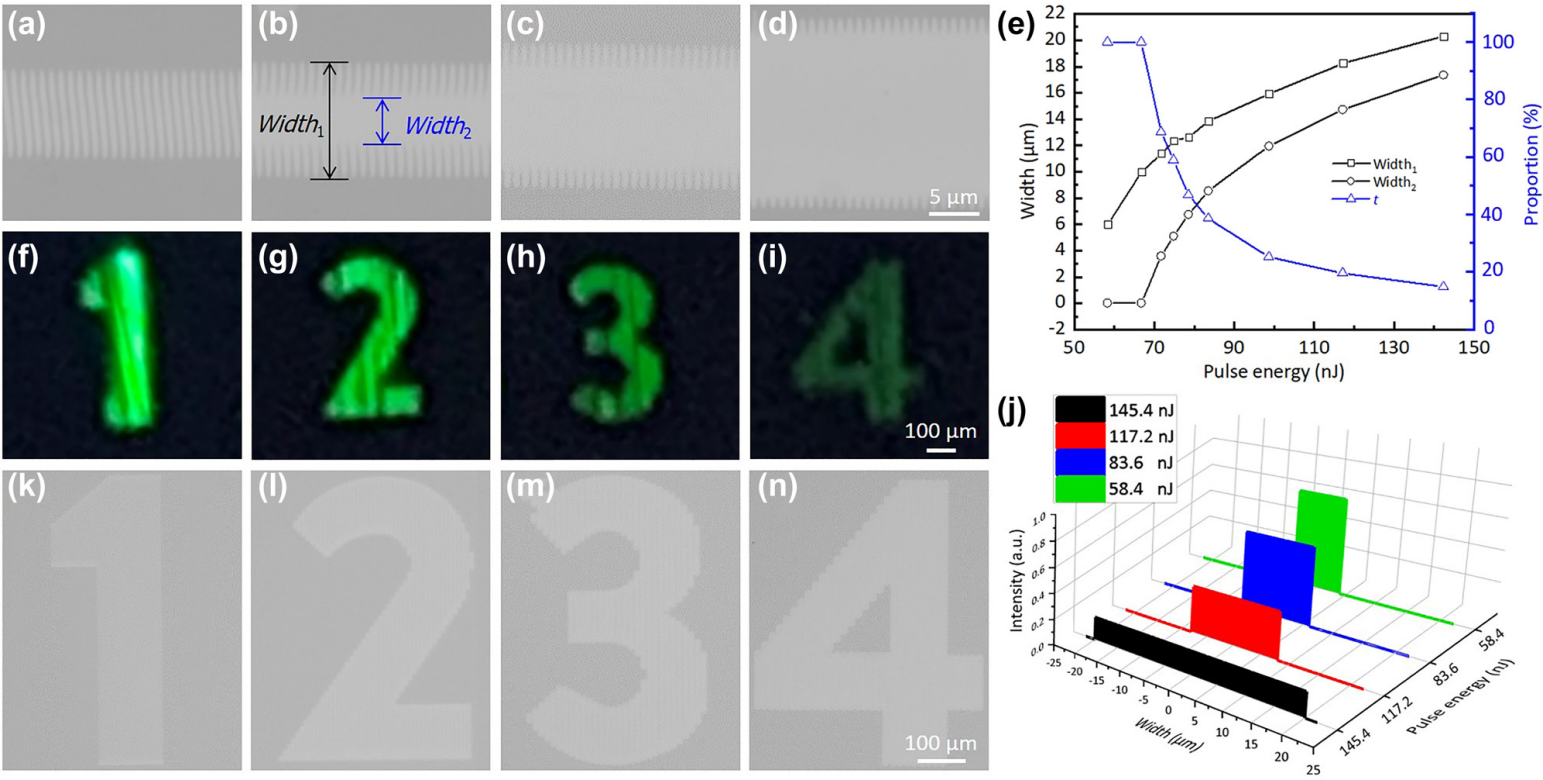
Optical microscopy images of laser scanned modified lines on GST surface with increased pulse energies. (a) 58.4, (b) 83.6, (c) 117.2, and (d) 145.4 nJ. The scanning speed is fixed at 500 μm/s. (e) The width of the scanned modified line, the width of the completely crystalized area, and the proportion (*t*) of the modified ripple structure as a function of the irradiated pulse energies. (f)–(i) Images of the four-level brightness captured by the camera under strong light irradiation, the patterns are composed by the modified structures shown in (a)–(d), respectively. (j) Measured average color intensities (normalized intensities) of the four patterns. (k)–(n) Optical images with corresponding four grayscale levels observed using an optical microscope.

**Figure 8:**
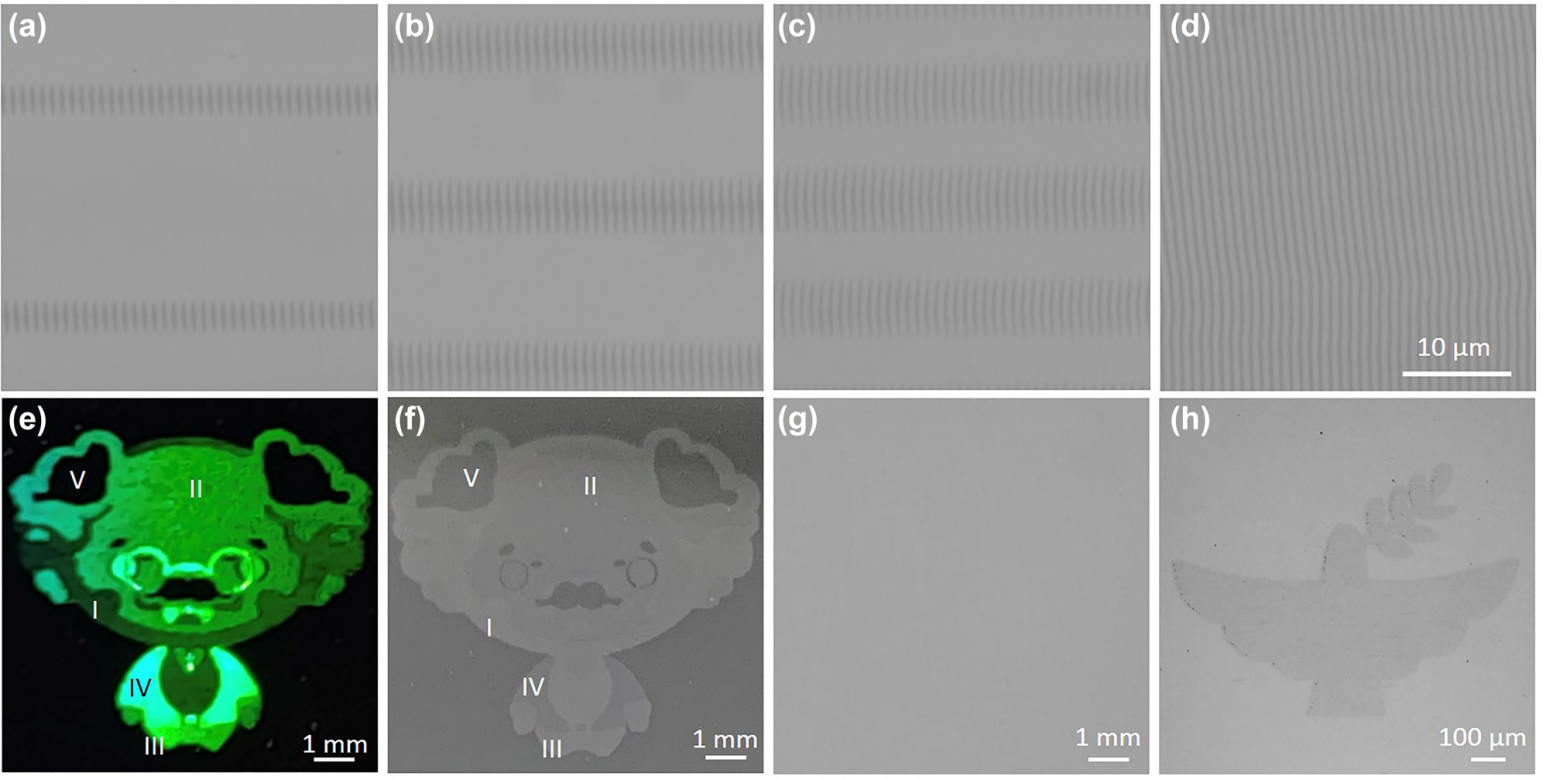
Multifunction and multilevel rewritable optical recording. (a)–(d) Optical microscopy images of line pixels with the pulse energy as 145.4, 117.2, 83.6 and 58.4 nJ, respectively, corresponding to regions I–IV in (e)–(g) written by the ps laser. (e) Five-brightness-level cartoon portrait of Albert Einstein captured by the camera under the illumination of strong light, and (f) depicts corresponding grayscale identification under natural light source. (g) Erased sample in crystalline phase state after thermal annealing. (h) Grayscale image rewritten by reamorphization.

Figure 7a–d illustrates the four types of ps-laser-induced modified surface structures with different structural grating proportions, under pulse energies of 58.4, 83.6, 117.2, and 145.4 nJ, respectively. As expectedly, modified ripple structures decorated with the completely crystalized area in the central laser scanning line can be observed, as shown in Figure 8b–d. The mechanism of this effect is shown schematically in Figure 6a. At high pulse energies, the central part of the Gaussian laser beam delivers a higher energy deposition to the GST film. The area whose deposited pulse intensity is higher above the crystallization threshold appears a different completely crystallized structure with respect to its peripheral region. This modified surface structures composed of the peripheral modified ripple structures and completely crystallized structures could be regarded as an intermediated modified structure transition from modified ripple structure to completely crystallized structure. As a matter of fact, one can note that the widths of each area could be changed by the pulse energies, and the proportion of the modified ripple structures decreased as the pulse energies increased. To numerically represent this ratio, we define a parameter *t* (modified ripple structure proportion) as: *t* = [(Width_1_ − Width_2_)/Width_1_] × 100%, where Width_1_ and Width_2_ are the line width of the scanned line and central completely crystallized structure, respectively. Figure 7e shows the dimensions and proportion of modified ripple structures as a function of irradiated pulse energies. It can be found that the dimensions (Width_1_ and Width_2_) were growing as the pulse energies increase. The parameter *t* decreased from 100 to 14.8% with the increase of pulse energy, which indicates the decreasing modified ripple structure ratio in the intermediated modified ripple structures. The 2D grating morphology of the modified ripple structures renders it’s a diffraction color effect. As the proportion of the 2D grating decreased, the diffraction intensity was dramatically weakened, which was accompanied by the brightness reduction. As can be demonstrated that, no diffraction colors can be observed for the completely crystallized structures.

As a demonstration, the aforementioned four types of the modified surface structures (Figure 7a–d) with different proportions of the modified ripple structure is used to write four patterns: numbers “1”, “2”, “3”, and “4”. The images captured by the camera of the four patterns under the exposure of strong light were shown in Figure 7f–i, which showed a structural color identification. As expected, the variation of the modified ripple structure proportion does directly affect the brightness. As can be seen, the brightness of the colored patterns (“1” to “4”) gradually reduced because of the decreasing proportion of modified ripple structure. The average color intensities (normalized intensities) measured for the four patterns also indicated that the intensity of brightness decreased with an increase in pulse energy, as displayed in Figure 7j. Results suggest that structural colors with multi-level brightness tones can be determined by deliberately tuning the laser parameters (pulse energy or scanning speed), which can be used for color image recording. As the patterns were illuminated by the natural light condition, no apparent structural color can be seen, and multi-level grayscale tones can be identified, as the optical images shown in Figure 7k–n. It can be observed that with increasing pulse energy, the color of the pattens becomes lighter due to the higher reflectivity. Two factors can be included: (1) The crystallinity of GST thin film was enhanced as the pulse energy increase, including its crystallization depth; (2) The area of crystalline GST increased as the proportion of the completely crystallized structures increases. The above results suggest that we can simultaneously tune the display of brightness and grayscale tones based on the integrated property–structure modulation of modified surface structures by tuning the irradiate laser parameters.

Further study was performed for the applications of real image recording with multi-level color brightness and grayscale tones. A five-brightness-level image and five-grayscale-level image of a cartoon portrait of Albert Einstein (Figure S3) were written into a GST medium by ps laser based on the aforementioned modified surface structures. Figure 8a–d displays processed areas of above four modified surface structures, respectively, correspond to region I–IV and the initially amorphous GST (unprocessed area) corresponds to region V in Figure 8e and f. The painted multilevel cartoon portrait of Albert Einstein consists of these four line pixels and unprocessed area. According to the diffraction effect of the grating, various colors throughout the visible spectrum were obtained by varying the incidence angle and viewing angle. We fixed the incidence angle (*α*) as 40° and changed the viewing angle (*β*) to explore the regularity of the color variation (Figure S4a). When the incidence angle is fixed, light of different wavelengths has different diffraction angles. In this study, various colors from red to purple were observed as the viewing angle gradually decreased. Figure 8e depicts the five-level brightness of the image of Einstein in green under the condition of strong light exposure (*α* = 40°) captured by the camera at 72° (*β*_2_). We can observe that the color became more and more bright from region I–IV and region V was dark because there was no geometric grating structure. Figure S4b and c shows the red and purple images obtained at viewing angles of 78° (*β*_1_) and 67° (*β*_2_), respectively. Because the processing area was large, the light intensity distribution in this area was uneven; thus, the brightness of the area closer to the incident light was higher. The five-level grayscale image of Einstein is depicted in Figure 8f. The gray color gradually became dark from region I–IV due to the decreasing optical reflectivity. However, the gray level range of GST is less adjustable when using line pixels than when using point pixels; thus, the grayscale image provided limited information. In a word, we successfully achieved multi-level brightness and grayscale image storage in the visible band through the integrated tuning of the surface morphology and optical reflectivity of GST.

Notably, the nonablated modified surface structures render the images can be erased by changing the selective crystallized regions back to homogeneous amorphized state. According to previous studies, one can erase selected crystallized areas of the prints using a single microsecond [47], nanosecond [29], picosecond [48], and femtosecond laser [49] pulse with an ultrahigh switching time. However, no convincing evidences have been demonstrated that the amorphization can be triggered by the continuous laser-scanning technique. This can be attributed to the quick quenching mechanism for the amorphization. Thus, in our studies, we erase the printed images through thermal annealing technique. By heating the entire sample on a hot plate to induce crystallization, the original color/grayscale patterns were gradually recovered, which erases all the image levels in parallel. Figure 8g shows the effect of the thermal annealing the printed sample at 250 °C for 20 min on a hot plate (detailed annealing process can be seen in Movie S1). As can be seen, the printed image was erased, making it possible to write a new image. However, it is worth noting that, as the amorphization can only happen in single pulse irradiation condition, the periodic modified ripple structures cannot be printed based on the crystallized GST film. Thus, only grayscale images coded by spatially-resolved amorphization can be achieved, as shown in Figure 8h. Furthermore, this reconfigurable functional surface also has the potential to repeatedly write and erase multiple images with color and grayscale identification onto the same region by change the thermal annealed sample to amorphous state (reamorphization). This reamorphization can be achieved by ns laser pulses [29], whose focused area could be large enough to amorphization the entire sample.

**Movie S1:** 

## 4 Conclusions

In this research, we introduced a flexible approach for ps laser-induced integrated property–structure modulation of GST in a one step. We successfully achieve the fabrication of surface micro/nanostructures with different phase states of GST by precisely controlling the distribution of laser–SPPs interference field. As a demonstration, we realized a multifunctional image storage with five-brightness structural colors and five-grayscale identification by ps laser. Subsequently, the image can be erased through thermal annealing and rewritten by single pulse irradiation. Our work thus lays the foundation for the high-quality and high-efficiency reconfigurable information storage by laser printing. We expect that the proposed approach of integrated property–structure modulation is useful for the fabrication and modulation of dynamic tunable metasurfaces, optical anticounterfeiting, and information storage.

## 5 Experimental section

*Sample preparation*: Initially amorphous GST thin films with a thickness of 200 nm were deposited on Si (100) substrates by using a radio-frequency magnetron sputtering system (Beijing Chuangshi Weina Technology, MSP-300B) with a high-purity stoichiometric target. Before sputtering, the Si substrates were cleaned using ethanol solution and then rinsed with deionized water under ultrasonic agitation to remove surface contamination. Sputtering was performed using Ar gas flow at room temperature under a pressure of 0.4 Pa, a power of 32 W, maintain a deposition rate of 0.6 nm s^−1^. The film thickness was confirmed using a 3D topography instrument (Wyko NT1100).

*Sample characterization*: The morphologies and microstructures of the fabricated structures were characterized through scanning electron microscopy (SEM; SU9000, HITACHI, Japan), transmission electron microscopy (TEM; JEM 2100F, JEOL, JAPAN), and atomic force microscopy (AFM; Dimension Edge PSS, Bruker, Germany). Raman spectra were recorded using a Raman spectroscope (Raman-11, Nanophoton, Japan), and X-ray diffraction (XRD) patterns were recorded in an angle interval of 20°–80° (2*θ*) by using an X-ray diffractometer (D/Max 2500 H, Rigaku, Japan).

*Numerical simulations*: Numerical simulations were conducted using a commercial software (Lumerical FDTD Solutions). The simulation region was terminated using periodic boundary conditions along the *x*-direction and *y*-direction of plane wave propagation and perfectly matched layer boundaries along the *z*-direction of plane wave propagation.

## Supplementary Material

Supplementary Material Details
