# Supplementary material for: Ultrafast laser-induced integrated property–structure modulation of Ge2Sb2Te5 for multifunction and multilevel rewritable optical recording
Source: Nanophotonics. 2022 May 17;11(13):3101–13. doi: 10.1515/nanoph-2022-0133 (PMC11501491; doi:10.1515/nanoph-2022-0133)
Supplement: Supplementary file 3 — Supplementary Material Details [file j_nanoph-2022-0133_suppl.docx]

**Supporting Information**

Kang Zhao, Weina Han*, Zihao Han, Xiaobin Zhang, Xingyi Zhang, Xiaofeng Duan, Mengmeng Wang, Yanping Yuan, and Pei Zuo

Ultrafast laser-induced integrated property–structure modulation of Ge_2_Sb_2_Te_5_ for multifunction and multilevel rewritable optical recording


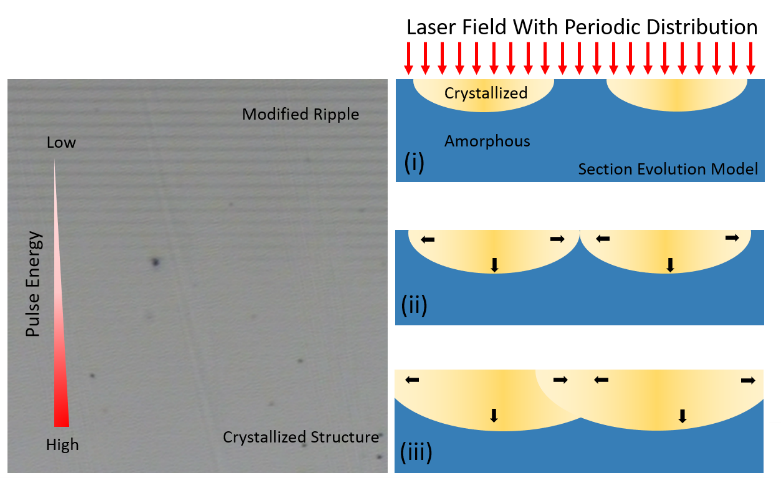


**Figure S1:** Schematic diagram of evolution mechanism from modified ripple structure to complete crystallization structure.

Figure S1 shows the schematic diagram of evolution mechanism from modified ripple structure to complete crystallization structure. The crystalline stripes will extend in width and depth with the increasing pulse energy, while the period of modified ripple structure will not change. When the width of the crystalline stripes will increase to be close to the periodic size, at this time, the adjacent crystalline stripes will be connected to form a complete crystalline structure on the surface.


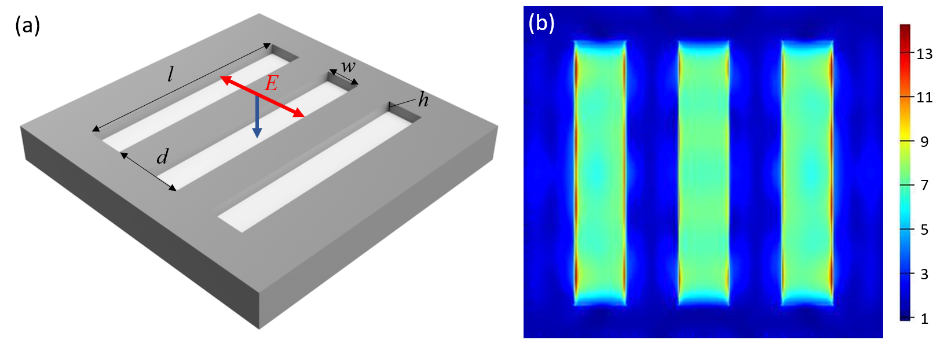


**Figure S2:** (a) Schematic modified ripple structure on the GST. The calculated electric field distributions of schematic structures (*l* = 3 μm, *w* = 0.55 μm, *d* = 0.55 μm and *h* = 0.1 μm) by horizontal laser polarization directions. E represents the laser polarization [orientation](javascript:;). (b) The calculated electric field distribution of the schematic structure.

**
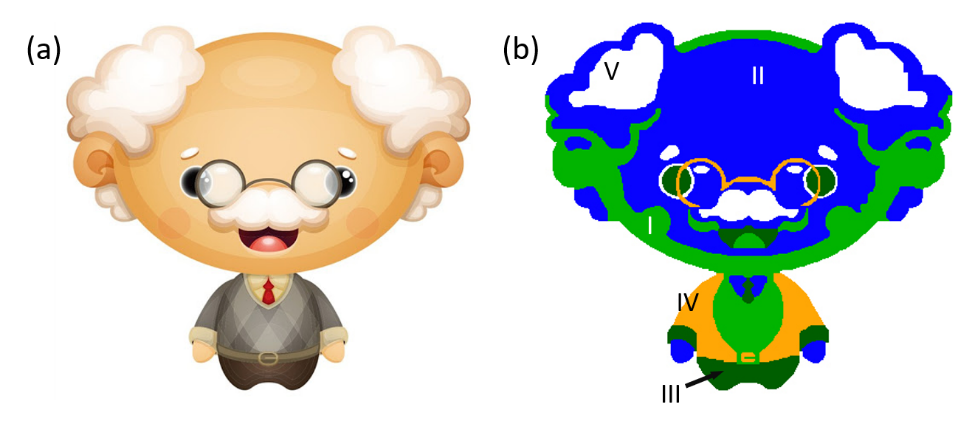
**

**Figure S3:** (a) Original cartoon portrait of Albert Einstein. (b) Cartoon portrait of Albert Einstein after processed by MATLAB.

Figure S3a is the original cartoon portrait of Albert Einstein. We process this original picture with MATLAB and divided into five regions according to the gray value, as shown in Figure S3b.


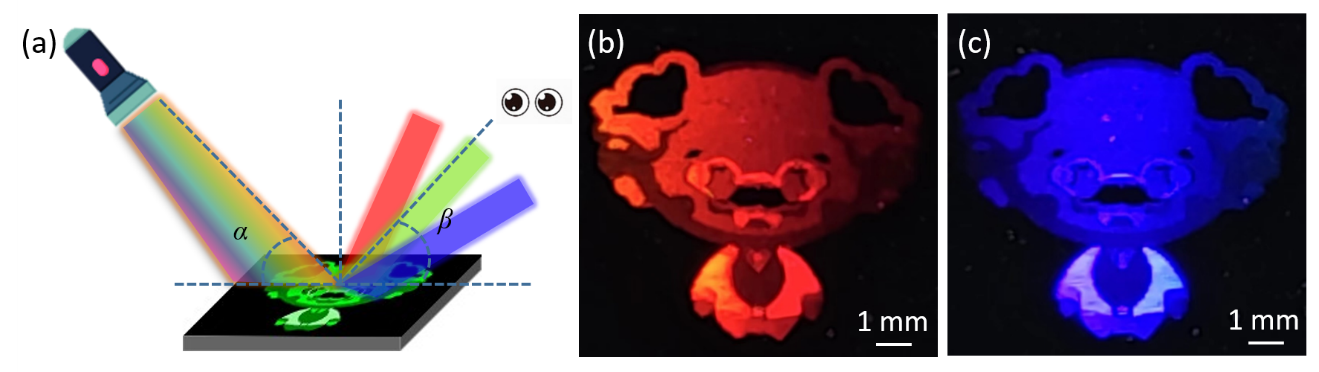


**Figure S4:** (a) The diagrammatic sketch of observation system under strong light illumination. (b) and (c) are four-brightness images based on structural colors under fixed angle (*α* = 40°) of strong light illumination captured by an ordinary camera at 78° (*β*_1_) and 67° (*β_3_*), respectively.

Figure S4a shows the diagrammatic sketch of observation system under strong light illumination. We fixed the incident light angle (*α*) as 40° and changed the viewing angle (*β*) to observe the color variation. Figure S4b and c show the red and blue image at viewing angle *β*_1_ = 78° and *β*_2_ = 67° photted by a camera, respectively.

**Movie S1.** We set the sample with cartoon portrait of Albert Einstein on the hot plate to erase the information by thermal annealing (at 250°C for 20 min). The process of erasure is recorded by a camera and the video was played at 50x speed.
